# Ethyl 2-(4-meth­oxy­phen­yl)-1-[3-(2-oxopyrrolidin-1-yl)prop­yl]-1*H*-benzimidazole-5-carboxyl­ate

**DOI:** 10.1107/S1600536811055966

**Published:** 2012-01-07

**Authors:** Yeong Keng Yoon, Mohamed Ashraf Ali, Tan Soo Choon, Safra Izuani Jama Asik, Ibrahim Abdul Razak

**Affiliations:** a Institute for Research in Molecular Medicine, Universiti Sains Malaysia, Minden 11800, Penang, Malaysia; b School of Physics, Universiti Sains Malaysia, 11800 USM, Penang, Malaysia

## Abstract

The asymmetric unit of the title compound, C_24_H_27_N_3_O_4_, contains two mol­ecules, *A* and *B*. The benzimidazole rings are essentially planar [maximum deviations = 0.0144 (10) and 0.0311 (8) Å in *A* and *B*, respectively]. The dihedral angle between the benzimidazole mean plane and its attached benzene ring is 36.90 (5) ° for mol­ecule *A* and 51.40 (5) ° for mol­ecule *B*. In both mol­ecules, the pyrrolidine ring adopts an envelope conformation with a C atom as the flap. In molecule *B*, the flap C atom is disordered over two positions in a 0.711 (6):0.289 (6) ratio. In the crystal, C—H⋯O inter­actions link the mol­ecules, generating [100] chains. The crystal packing also features weak π–π inter­actions between the imidazole and benzene rings [centroid–centroid distances = 3.8007 (7) and 3.8086 (7) Å] and between the benzene rings [centroid–centroid distance = 3.7001 (7) Å] and C—H⋯π inter­actions involving the benzene rings.

## Related literature

For the biological activity of benzimidazole derivatives, see: Spasov *et al.* (1999 ▶); Tanious *et al.* (2004) ▶; Townsend & Revankar (1970 ▶). For ring conformations, see: Cremer & Pople (1975 ▶). For the stability of the temperature controller used in the data collection, see: Cosier & Glazer (1986 ▶). For hydrogen-bond motifs, see: Bernstein *et al.* (1995 ▶).
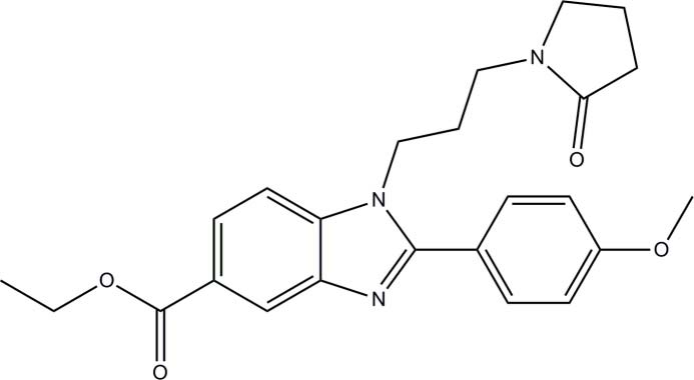


## Experimental

### 

#### Crystal data

C_24_H_27_N_3_O_4_
*M* *_r_* = 421.49
Triclinic, 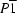

*a* = 10.7455 (3) Å
*b* = 12.2235 (3) Å
*c* = 16.1967 (4) Å
α = 86.162 (1)°
β = 80.917 (1)°
γ = 88.275 (1)°
*V* = 2095.60 (9) Å^3^
*Z* = 4
Mo *K*α radiation
μ = 0.09 mm^−1^
*T* = 100 K
0.74 × 0.43 × 0.14 mm

#### Data collection

Bruker SMART APEXII CCD diffractometer
Absorption correction: multi-scan (*SADABS*; Bruker, 2009 ▶) *T*_min_ = 0.935, *T*_max_ = 0.987
47137 measured reflections
11119 independent reflections
9491 reflections with *I* > 2σ(*I*)
*R*_int_ = 0.026

#### Refinement

*R*[*F* ^2^ > 2σ(*F* ^2^)] = 0.044
*wR*(*F* ^2^) = 0.133
*S* = 1.02
11119 reflections
573 parameters
H-atom parameters constrained
Δρ_max_ = 0.46 e Å^−3^
Δρ_min_ = −0.27 e Å^−3^

### 

Data collection: *APEX2* (Bruker, 2009 ▶); cell refinement: *SAINT* (Bruker, 2009 ▶); data reduction: *SAINT*; program(s) used to solve structure: *SHELXTL* (Sheldrick, 2008 ▶); program(s) used to refine structure: *SHELXTL*; molecular graphics: *SHELXTL*; software used to prepare material for publication: *SHELXTL* and *PLATON* (Spek, 2009 ▶).

## Supplementary Material

Crystal structure: contains datablock(s) global, I. DOI: 10.1107/S1600536811055966/hb6575sup1.cif

Structure factors: contains datablock(s) I. DOI: 10.1107/S1600536811055966/hb6575Isup2.hkl

Supplementary material file. DOI: 10.1107/S1600536811055966/hb6575Isup3.cml

Additional supplementary materials:  crystallographic information; 3D view; checkCIF report

## Figures and Tables

**Table 1:** Hydrogen-bond geometry (Å, °) *Cg*1, *Cg*2, *Cg*4, *Cg*5 and *Cg*6 are the centroids of the N1*B*–N2*B*/C1*B*/C6*B*–C7*B*, C1*A*–C6*A*, C1*B*–C6*B*, C8*B*–C13*B* and C8*A*–C13*A* rings, respectively.

*D*—H⋯*A*	*D*—H	H⋯*A*	*D*⋯*A*	*D*—H⋯*A*
C5*A*—H5*AA*⋯O3*B*^i^	0.95	2.30	3.2382 (15)	171
C5*B*—H5*BA*⋯O3*A*^ii^	0.95	2.38	3.3244 (15)	172
C19*A*—H19*A*⋯O2*A*^i^	0.99	2.58	3.3034 (14)	130
C19*A*—H19*B*⋯O3*B*^i^	0.99	2.53	3.2681 (15)	131
C19*B*—H19*C*⋯O3*A*^ii^	0.99	2.46	3.2007 (15)	131
C9*B*—H9*BA*⋯*Cg*1^iii^	0.95	2.85	3.5237 (12)	129
C10*B*—H10*B*⋯*Cg*4^iii^	0.95	2.80	3.4556 (12)	127
C16*A*—H16*B*⋯*Cg*5	0.98	2.83	3.8093 (15)	175
C18*A*—H18*A*⋯*Cg*6^iv^	0.99	2.70	3.5251 (12)	141
C24*A*—H24*C*⋯*Cg*2^iv^	0.98	2.76	3.7186 (14)	165

Symmetry codes: (i) 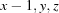; (ii) 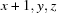; (iii) 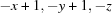; (iv) 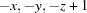.

**Fig. 1.**
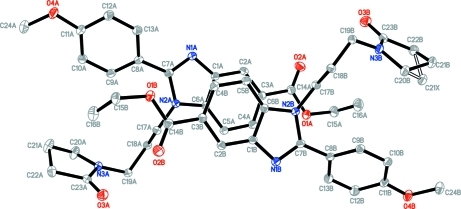
The structure of the title compound, showing 50% probability displacement ellipsoids. Hydrogen atoms are omitted for clarity.

**Fig. 2.**
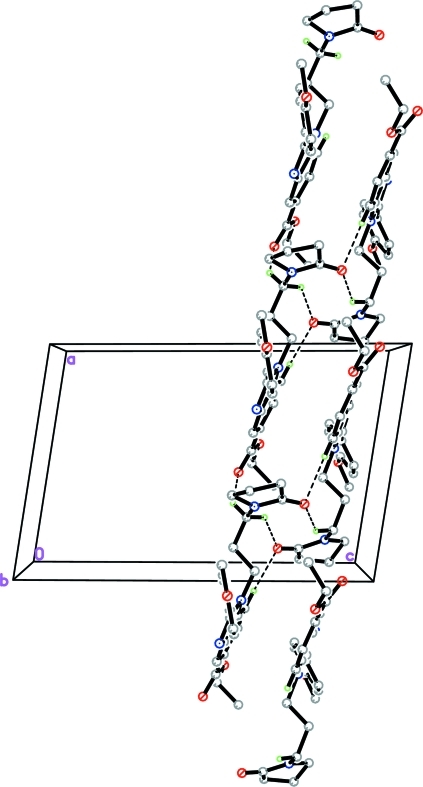
The crystal packing, viewed along the b-axis, showing the molecules are connected into ribbon along a axis. Hydrogen bonds are shown as dashed lines.

## supplementary crystallographic information

### Comment

The benzimidazole nucleus is an important pharmacophore in drug discovery (Spasov *et al.*, 1999). Substituted benzimidazole is the key building block for numerous compounds which plays crucial roles in the function of biologically important molecules (Tanious *et al.*, 2004). In particular, substituted benzimidazoles are recognized as potential anticancer agents (Townsend & Revankar, 1970). In view of their importance, the crystal structure determination of the title compound was carried out and the results are presented here.

The asymmetric unit of the title compound consist two crystallographically independent molecules (A and B). The benzimidazoles N1A–N2A/C1A–C7A and N1B–N2B/C1B–C7B rings are essentially planar with maximum deviations of 0.0144 (10) and 0.0311 (8) Å at atoms N1A and C3B, respectively. In molecule A, the dihedral angle between the mean plane through the benzimidazole, (N1A–N2A/C1A–C7A) ring with the mean plane through the benzene, (C8A–C13A) ring is 36.90 (5) °. In molecule B, the dihedral angle between the corresponding rings is 51.40 (5) °. Atom C21B is disordered (Fig. 2) over two positions, with occupancy ratios of 0.711 (6):0.289 (6). The pyrrolidin moiety in both molecules adopts an envelope conformation. In molecule A, the puckering parameters Q = 0.3051 (14) Å and φ = 245.2 (2)° with C21A at the flap. In molecule B, the puckering parameters for the disordered pyrrolidin ring are Q = 0.2696 (16) Å and φ = 247.9 (3)° with C21B at the flap and Q = 0.248 (3) Å and φ = 78.7 (4)° with C21X at the flap (Cremer & Pople, 1975).

In the crystal packing (Fig. 2), R^2^_2_(10) ring motifs (Bernstein *et al.*, 1995) are formed by C19A—H19B···O3B (x-1,y,z) and C19B—H19C···O3A(1+x,y,z) intermolecular interactions. C5A—H5AA···O3B(x-1,y,z), C5B—H5BA···O3A(1+x,y,z) and C19A—H19A···O2A (x-1,y,z) interactions further link the molecules into ribbon along the *a* axis. π–π interactions are observed within the benzimidazole rings system between the imidazole, (N1B–N2B/C1B/C6B–C7B; centroid *Cg*1 and N1A–N2A/C1A/C6A–C7A; centroid *Cg*3) and the benzene, (C1A–C6A;centroid *Cg*2 and C1B–C6B;centroid *Cg*4) rings with *Cg*1···*Cg*2 distance of 3.8007 (7) Å and *Cg*3···*Cg*4 distance of 3.8085 (7) Å. π–π interactions are also observed between the benzene rings with a *Cg*4···*Cg*2 distance of 3.7001 (7) Å. The crystal packing are further stabilized by weak C—H···π interactions (Table 1) involving benzimidazole and benzene rings.

### Experimental

Ethyl 3-amino-4-(3(2-oxopyrrolidin-1yl)propylamino)benzoate (0.84 mmol) and sodium metabisulfite adduct of 4-methoxybenzaldehyde (1.68 mmol) were dissolved in DMF. The reaction mixture was reflux at 130 °C for 2 hrs. After completion, the reaction mixture was diluted in Ethyl acetate (20 mL) and washed with water (20 mL). The organic layer was collected, dried over Na_2_SO_4_ and the evaporated in vacuo to yield the product. The product was recrystallised from ethyl acetate to form yellow plates.

### Refinement

Atom C21B is disodered over two positions, with occupancy ratios of 0.711 (6):0.289 (6). All the H atoms positioned geometrically and refined using a riding model with with C–H = 0.95–0.99 Å. The *U*_iso_ values were constrained to be 1.5*U*_eq_ (methyl-H atom) and 1.2*U*_eq_ (other H atoms). The rotating model group was applied for the methyl groups.

### Crystal data

**Table:**

C_24_H_27_N_3_O_4_	*Z* = 4
*M**_r_* = 421.49	*F*(000) = 896
Triclinic, *P*1	*D*_x_ = 1.336 Mg m^−^^3^
Hall symbol: -P 1	Mo *K*α radiation, λ = 0.71073 Å
*a* = 10.7455 (3) Å	Cell parameters from 9863 reflections
*b* = 12.2235 (3) Å	θ = 2.5–32.8°
*c* = 16.1967 (4) Å	µ = 0.09 mm^−^^1^
α = 86.162 (1)°	*T* = 100 K
β = 80.917 (1)°	Plate, yellow
γ = 88.275 (1)°	0.74 × 0.43 × 0.14 mm
*V* = 2095.60 (9) Å^3^	

### Data collection

**Table:**

Bruker SMART APEXII CCD diffractometer	11119 independent reflections
Radiation source: fine-focus sealed tube	9491 reflections with *I* > 2σ(*I*)
graphite	*R*_int_ = 0.026
φ and ω scans	θ_max_ = 29.0°, θ_min_ = 1.3°
Absorption correction: multi-scan (*SADABS*; Bruker, 2009)	*h* = −14→14
*T*_min_ = 0.935, *T*_max_ = 0.987	*k* = −16→16
47137 measured reflections	*l* = −22→22

### Refinement

**Table:**

Refinement on *F*^2^	Primary atom site location: structure-invariant direct methods
Least-squares matrix: full	Secondary atom site location: difference Fourier map
*R*[*F*^2^ > 2σ(*F*^2^)] = 0.044	Hydrogen site location: inferred from neighbouring sites
*wR*(*F*^2^) = 0.133	H-atom parameters constrained
*S* = 1.02	*w* = 1/[σ^2^(*F*_o_^2^) + (0.0777*P*)^2^ + 0.709*P*] where *P* = (*F*_o_^2^ + 2*F*_c_^2^)/3
11119 reflections	(Δ/σ)_max_ < 0.001
573 parameters	Δρ_max_ = 0.46 e Å^−^^3^
0 restraints	Δρ_min_ = −0.27 e Å^−^^3^

### Special details

**Table:**

Experimental. The crystal was placed in the cold stream of an Oxford Cryosystems Cobra open-flow nitrogen cryostat (Cosier & Glazer, 1986) operating at 100.0 (1) K.
Geometry. All esds (except the esd in the dihedral angle between two l.s. planes) are estimated using the full covariance matrix. The cell esds are taken into account individually in the estimation of esds in distances, angles and torsion angles; correlations between esds in cell parameters are only used when they are defined by crystal symmetry. An approximate (isotropic) treatment of cell esds is used for estimating esds involving l.s. planes.
Refinement. Refinement of F^2^ against ALL reflections. The weighted R-factor wR and goodness of fit S are based on F^2^, conventional R-factors R are based on F, with F set to zero for negative F^2^. The threshold expression of F^2^ > 2sigma(F^2^) is used only for calculating R-factors(gt) etc. and is not relevant to the choice of reflections for refinement. R-factors based on F^2^ are statistically about twice as large as those based on F, and R- factors based on ALL data will be even larger.

### Fractional atomic coordinates and isotropic or equivalent isotropic displacement parameters (Å^2^)

**Table:**

	*x*	*y*	*z*	*U*_iso_*/*U*_eq_	Occ. (<1)
O1A	0.43138 (8)	0.57270 (7)	0.36306 (6)	0.0251 (2)	
O2A	0.55442 (8)	0.43980 (7)	0.41340 (6)	0.02447 (19)	
O3A	−0.31842 (9)	0.15173 (7)	0.21854 (6)	0.02455 (19)	
O4A	0.02719 (8)	−0.37276 (7)	0.40345 (6)	0.02395 (19)	
N1A	0.27573 (9)	0.09583 (8)	0.39577 (6)	0.01564 (19)	
N2A	0.09660 (8)	0.16243 (7)	0.35276 (6)	0.01339 (18)	
N3A	−0.30856 (9)	0.11955 (8)	0.35853 (6)	0.01669 (19)	
C1A	0.28242 (10)	0.20905 (9)	0.38208 (7)	0.0144 (2)	
C2A	0.37755 (10)	0.28003 (9)	0.39205 (7)	0.0164 (2)	
H2AA	0.4525	0.2530	0.4112	0.020*	
C3A	0.35928 (10)	0.39193 (9)	0.37303 (7)	0.0157 (2)	
C4A	0.24819 (10)	0.43191 (9)	0.34413 (7)	0.0159 (2)	
H4AA	0.2390	0.5083	0.3308	0.019*	
C5A	0.15220 (10)	0.36284 (9)	0.33465 (7)	0.0149 (2)	
H5AA	0.0773	0.3899	0.3155	0.018*	
C6A	0.17139 (10)	0.25157 (9)	0.35475 (7)	0.0134 (2)	
C7A	0.16384 (10)	0.07135 (9)	0.37803 (7)	0.0144 (2)	
C8A	0.11945 (10)	−0.04209 (9)	0.38429 (7)	0.0150 (2)	
C9A	−0.00483 (10)	−0.07252 (9)	0.41281 (7)	0.0165 (2)	
H9AA	−0.0670	−0.0172	0.4273	0.020*	
C10A	−0.04016 (11)	−0.18211 (9)	0.42064 (7)	0.0169 (2)	
H10A	−0.1252	−0.2009	0.4405	0.020*	
C11A	0.05013 (11)	−0.26363 (9)	0.39907 (7)	0.0174 (2)	
C12A	0.17569 (11)	−0.23495 (10)	0.37145 (8)	0.0207 (2)	
H12A	0.2378	−0.2905	0.3575	0.025*	
C13A	0.20951 (11)	−0.12620 (9)	0.36439 (8)	0.0186 (2)	
H13A	0.2951	−0.1078	0.3458	0.022*	
C14A	0.45920 (11)	0.46763 (9)	0.38589 (8)	0.0184 (2)	
C15A	0.52194 (12)	0.65402 (10)	0.37453 (9)	0.0266 (3)	
H15A	0.5595	0.6327	0.4254	0.032*	
H15B	0.4785	0.7260	0.3825	0.032*	
C16A	0.62410 (13)	0.66355 (11)	0.29988 (9)	0.0290 (3)	
H16A	0.6802	0.7230	0.3064	0.043*	
H16B	0.5863	0.6794	0.2490	0.043*	
H16C	0.6725	0.5944	0.2954	0.043*	
C17A	−0.02866 (10)	0.17174 (9)	0.32769 (7)	0.0142 (2)	
H17A	−0.0535	0.0991	0.3129	0.017*	
H17B	−0.0257	0.2228	0.2774	0.017*	
C18A	−0.12686 (10)	0.21337 (9)	0.39798 (7)	0.0153 (2)	
H18A	−0.1299	0.1619	0.4481	0.018*	
H18B	−0.1012	0.2856	0.4130	0.018*	
C19A	−0.25791 (10)	0.22453 (9)	0.37307 (8)	0.0168 (2)	
H19A	−0.3158	0.2593	0.4180	0.020*	
H19B	−0.2539	0.2733	0.3214	0.020*	
C20A	−0.35991 (13)	0.04285 (10)	0.42742 (8)	0.0249 (3)	
H20A	−0.4395	0.0717	0.4586	0.030*	
H20B	−0.2989	0.0268	0.4667	0.030*	
C21A	−0.38274 (14)	−0.05892 (10)	0.38240 (8)	0.0273 (3)	
H21A	−0.3070	−0.1076	0.3755	0.033*	
H21B	−0.4549	−0.1007	0.4134	0.033*	
C22A	−0.41136 (12)	−0.01174 (10)	0.29818 (8)	0.0224 (2)	
H22A	−0.5030	0.0015	0.2998	0.027*	
H22B	−0.3806	−0.0617	0.2534	0.027*	
C23A	−0.34068 (10)	0.09538 (9)	0.28431 (8)	0.0170 (2)	
C24A	−0.09938 (12)	−0.40763 (10)	0.43130 (8)	0.0243 (3)	
H24A	−0.1026	−0.4875	0.4300	0.037*	
H24B	−0.1550	−0.3729	0.3943	0.037*	
H24C	−0.1271	−0.3863	0.4887	0.037*	
O1B	0.11742 (8)	0.00048 (7)	0.14833 (6)	0.02285 (19)	
O2B	0.00913 (9)	0.12746 (7)	0.07933 (6)	0.0249 (2)	
O3B	0.89232 (9)	0.42845 (7)	0.26430 (6)	0.02442 (19)	
O4B	0.52357 (8)	0.94651 (7)	0.10398 (6)	0.02073 (18)	
N1B	0.26036 (9)	0.47944 (7)	0.11609 (6)	0.01480 (18)	
N2B	0.44823 (8)	0.41675 (7)	0.14796 (6)	0.01313 (18)	
N3B	0.85576 (9)	0.46961 (8)	0.12995 (6)	0.01700 (19)	
C1B	0.25893 (10)	0.36593 (9)	0.12809 (7)	0.0135 (2)	
C2B	0.16548 (10)	0.29273 (9)	0.11952 (7)	0.0143 (2)	
H2BA	0.0873	0.3181	0.1042	0.017*	
C3B	0.19026 (10)	0.18126 (9)	0.13407 (7)	0.0144 (2)	
C4B	0.30537 (10)	0.14328 (9)	0.15872 (7)	0.0150 (2)	
H4BA	0.3179	0.0670	0.1707	0.018*	
C5B	0.40013 (10)	0.21455 (9)	0.16585 (7)	0.0148 (2)	
H5BA	0.4780	0.1891	0.1815	0.018*	
C6B	0.37524 (10)	0.32567 (9)	0.14884 (7)	0.0130 (2)	
C7B	0.37424 (10)	0.50635 (9)	0.12798 (7)	0.0137 (2)	
C8B	0.41785 (10)	0.61994 (9)	0.12023 (7)	0.0141 (2)	
C9B	0.53045 (10)	0.65103 (9)	0.07059 (7)	0.0154 (2)	
H9BA	0.5820	0.5969	0.0416	0.018*	
C10B	0.56981 (10)	0.75954 (9)	0.06227 (7)	0.0156 (2)	
H10B	0.6469	0.7791	0.0279	0.019*	
C11B	0.49422 (11)	0.83879 (9)	0.10526 (7)	0.0161 (2)	
C12B	0.37896 (11)	0.80928 (9)	0.15378 (8)	0.0208 (2)	
H12B	0.3266	0.8636	0.1820	0.025*	
C13B	0.34122 (11)	0.70155 (9)	0.16080 (8)	0.0192 (2)	
H13B	0.2626	0.6825	0.1934	0.023*	
C14B	0.09513 (11)	0.10324 (9)	0.11753 (7)	0.0166 (2)	
C15B	0.03339 (12)	−0.08339 (10)	0.13095 (9)	0.0244 (3)	
H15C	0.0753	−0.1563	0.1352	0.029*	
H15D	0.0149	−0.0700	0.0731	0.029*	
C16B	−0.08748 (13)	−0.08261 (11)	0.19151 (9)	0.0287 (3)	
H16D	−0.1410	−0.1416	0.1802	0.043*	
H16E	−0.1314	−0.0119	0.1850	0.043*	
H16F	−0.0690	−0.0938	0.2489	0.043*	
C17B	0.57713 (10)	0.41302 (9)	0.16718 (7)	0.0143 (2)	
H17C	0.5806	0.3642	0.2181	0.017*	
H17D	0.6005	0.4874	0.1791	0.017*	
C18B	0.67204 (10)	0.37172 (9)	0.09507 (7)	0.0149 (2)	
H18C	0.6703	0.4219	0.0447	0.018*	
H18D	0.6469	0.2984	0.0819	0.018*	
C19B	0.80624 (10)	0.36410 (9)	0.11548 (7)	0.0162 (2)	
H19C	0.8075	0.3142	0.1661	0.019*	
H19D	0.8623	0.3316	0.0685	0.019*	
C20B	0.86896 (11)	0.56181 (10)	0.06720 (8)	0.0199 (2)	
H20C	0.7880	0.6024	0.0663	0.024*	0.711 (6)
H20D	0.9005	0.5372	0.0106	0.024*	0.711 (6)
H20E	0.7929	0.5723	0.0433	0.024*	0.289 (6)
H20F	0.9370	0.5472	0.0230	0.024*	0.289 (6)
C21B	0.9682 (2)	0.63305 (15)	0.09897 (11)	0.0199 (5)	0.711 (6)
H21C	1.0547	0.6143	0.0720	0.024*	0.711 (6)
H21D	0.9514	0.7122	0.0870	0.024*	0.711 (6)
C21X	0.8945 (5)	0.6602 (4)	0.1078 (3)	0.0220 (13)	0.289 (6)
H21E	0.8165	0.7037	0.1245	0.026*	0.289 (6)
H21F	0.9569	0.7070	0.0711	0.026*	0.289 (6)
C22B	0.95215 (12)	0.60541 (10)	0.19016 (8)	0.0227 (2)	
H22C	0.8937	0.6584	0.2208	0.027*	0.711 (6)
H22D	1.0341	0.6058	0.2107	0.027*	0.711 (6)
H22E	1.0426	0.6022	0.1796	0.027*	0.289 (6)
H22F	0.9250	0.6462	0.2386	0.027*	0.289 (6)
C23B	0.89772 (10)	0.49098 (9)	0.20152 (8)	0.0176 (2)	
C24B	0.64097 (12)	0.98164 (10)	0.05625 (8)	0.0232 (2)	
H24D	0.6531	1.0590	0.0648	0.035*	
H24E	0.6399	0.9728	−0.0033	0.035*	
H24F	0.7101	0.9372	0.0746	0.035*	

### Atomic displacement parameters (Å^2^)

**Table:**

	*U*^11^	*U*^22^	*U*^33^	*U*^12^	*U*^13^	*U*^23^
O1A	0.0202 (4)	0.0133 (4)	0.0431 (6)	−0.0036 (3)	−0.0094 (4)	−0.0003 (4)
O2A	0.0184 (4)	0.0217 (4)	0.0351 (5)	0.0006 (3)	−0.0088 (4)	−0.0047 (4)
O3A	0.0289 (5)	0.0235 (4)	0.0225 (5)	−0.0046 (3)	−0.0100 (4)	0.0054 (3)
O4A	0.0241 (4)	0.0126 (4)	0.0340 (5)	−0.0021 (3)	−0.0001 (4)	−0.0035 (3)
N1A	0.0153 (4)	0.0134 (4)	0.0182 (5)	0.0009 (3)	−0.0029 (3)	−0.0001 (3)
N2A	0.0130 (4)	0.0119 (4)	0.0152 (4)	0.0000 (3)	−0.0021 (3)	−0.0003 (3)
N3A	0.0168 (4)	0.0146 (4)	0.0183 (5)	−0.0031 (3)	−0.0025 (4)	0.0019 (3)
C1A	0.0140 (5)	0.0131 (5)	0.0156 (5)	0.0013 (4)	−0.0014 (4)	−0.0006 (4)
C2A	0.0129 (5)	0.0168 (5)	0.0196 (5)	0.0011 (4)	−0.0033 (4)	−0.0011 (4)
C3A	0.0141 (5)	0.0146 (5)	0.0183 (5)	−0.0007 (4)	−0.0014 (4)	−0.0020 (4)
C4A	0.0162 (5)	0.0137 (5)	0.0174 (5)	0.0003 (4)	−0.0016 (4)	−0.0003 (4)
C5A	0.0153 (5)	0.0136 (5)	0.0159 (5)	0.0010 (4)	−0.0028 (4)	0.0000 (4)
C6A	0.0131 (5)	0.0137 (5)	0.0129 (5)	−0.0007 (4)	−0.0007 (4)	−0.0009 (4)
C7A	0.0157 (5)	0.0133 (5)	0.0135 (5)	0.0012 (4)	−0.0008 (4)	−0.0005 (4)
C8A	0.0170 (5)	0.0135 (5)	0.0141 (5)	0.0012 (4)	−0.0024 (4)	0.0002 (4)
C9A	0.0170 (5)	0.0142 (5)	0.0173 (5)	0.0023 (4)	−0.0008 (4)	0.0001 (4)
C10A	0.0168 (5)	0.0170 (5)	0.0163 (5)	−0.0001 (4)	−0.0014 (4)	0.0005 (4)
C11A	0.0211 (5)	0.0135 (5)	0.0174 (5)	−0.0009 (4)	−0.0025 (4)	−0.0009 (4)
C12A	0.0199 (5)	0.0146 (5)	0.0266 (6)	0.0023 (4)	0.0002 (4)	−0.0046 (4)
C13A	0.0171 (5)	0.0157 (5)	0.0221 (6)	0.0010 (4)	0.0002 (4)	−0.0023 (4)
C14A	0.0171 (5)	0.0159 (5)	0.0218 (6)	−0.0006 (4)	−0.0005 (4)	−0.0039 (4)
C15A	0.0232 (6)	0.0175 (6)	0.0407 (8)	−0.0054 (4)	−0.0075 (5)	−0.0057 (5)
C16A	0.0299 (7)	0.0263 (7)	0.0319 (7)	−0.0080 (5)	−0.0067 (5)	−0.0025 (5)
C17A	0.0134 (5)	0.0145 (5)	0.0152 (5)	0.0002 (4)	−0.0034 (4)	−0.0006 (4)
C18A	0.0151 (5)	0.0136 (5)	0.0174 (5)	0.0006 (4)	−0.0032 (4)	−0.0016 (4)
C19A	0.0145 (5)	0.0133 (5)	0.0228 (6)	0.0002 (4)	−0.0040 (4)	−0.0003 (4)
C20A	0.0349 (7)	0.0192 (6)	0.0188 (6)	−0.0076 (5)	0.0016 (5)	0.0020 (4)
C21A	0.0393 (7)	0.0172 (6)	0.0239 (6)	−0.0076 (5)	0.0004 (5)	0.0007 (5)
C22A	0.0207 (6)	0.0207 (6)	0.0264 (6)	−0.0060 (4)	−0.0046 (5)	−0.0006 (5)
C23A	0.0127 (5)	0.0170 (5)	0.0218 (6)	0.0000 (4)	−0.0047 (4)	0.0009 (4)
C24A	0.0275 (6)	0.0192 (6)	0.0255 (6)	−0.0067 (5)	−0.0003 (5)	−0.0015 (5)
O1B	0.0219 (4)	0.0138 (4)	0.0349 (5)	−0.0040 (3)	−0.0111 (4)	0.0006 (3)
O2B	0.0248 (4)	0.0216 (4)	0.0311 (5)	−0.0033 (3)	−0.0136 (4)	0.0008 (4)
O3B	0.0269 (5)	0.0238 (4)	0.0242 (5)	−0.0018 (3)	−0.0112 (4)	0.0039 (3)
O4B	0.0247 (4)	0.0118 (4)	0.0246 (4)	−0.0027 (3)	0.0000 (3)	−0.0020 (3)
N1B	0.0148 (4)	0.0128 (4)	0.0164 (5)	0.0003 (3)	−0.0018 (3)	0.0003 (3)
N2B	0.0123 (4)	0.0112 (4)	0.0157 (4)	0.0003 (3)	−0.0022 (3)	0.0001 (3)
N3B	0.0186 (4)	0.0141 (4)	0.0185 (5)	−0.0044 (3)	−0.0039 (4)	0.0021 (3)
C1B	0.0132 (5)	0.0135 (5)	0.0132 (5)	0.0010 (4)	−0.0011 (4)	0.0002 (4)
C2B	0.0129 (5)	0.0147 (5)	0.0148 (5)	0.0006 (4)	−0.0015 (4)	0.0000 (4)
C3B	0.0143 (5)	0.0147 (5)	0.0140 (5)	−0.0009 (4)	−0.0012 (4)	−0.0008 (4)
C4B	0.0163 (5)	0.0129 (5)	0.0155 (5)	0.0004 (4)	−0.0023 (4)	−0.0004 (4)
C5B	0.0142 (5)	0.0139 (5)	0.0159 (5)	0.0015 (4)	−0.0023 (4)	0.0003 (4)
C6B	0.0128 (5)	0.0137 (5)	0.0123 (5)	−0.0003 (4)	−0.0013 (4)	−0.0005 (4)
C7B	0.0142 (5)	0.0129 (5)	0.0134 (5)	0.0017 (4)	−0.0013 (4)	0.0000 (4)
C8B	0.0150 (5)	0.0123 (5)	0.0152 (5)	0.0008 (4)	−0.0033 (4)	0.0004 (4)
C9B	0.0171 (5)	0.0132 (5)	0.0152 (5)	0.0014 (4)	−0.0011 (4)	−0.0011 (4)
C10B	0.0164 (5)	0.0146 (5)	0.0153 (5)	−0.0007 (4)	−0.0019 (4)	0.0008 (4)
C11B	0.0195 (5)	0.0116 (5)	0.0177 (5)	−0.0003 (4)	−0.0045 (4)	−0.0004 (4)
C12B	0.0214 (5)	0.0141 (5)	0.0252 (6)	0.0026 (4)	0.0020 (5)	−0.0033 (4)
C13B	0.0166 (5)	0.0161 (5)	0.0232 (6)	0.0010 (4)	0.0018 (4)	−0.0012 (4)
C14B	0.0173 (5)	0.0151 (5)	0.0172 (5)	−0.0007 (4)	−0.0017 (4)	−0.0015 (4)
C15B	0.0236 (6)	0.0158 (5)	0.0356 (7)	−0.0048 (4)	−0.0091 (5)	−0.0035 (5)
C16B	0.0305 (7)	0.0244 (6)	0.0314 (7)	−0.0083 (5)	−0.0037 (5)	−0.0019 (5)
C17B	0.0127 (5)	0.0157 (5)	0.0150 (5)	−0.0002 (4)	−0.0041 (4)	−0.0002 (4)
C18B	0.0141 (5)	0.0145 (5)	0.0162 (5)	0.0002 (4)	−0.0031 (4)	−0.0010 (4)
C19B	0.0136 (5)	0.0132 (5)	0.0220 (6)	−0.0010 (4)	−0.0039 (4)	−0.0006 (4)
C20B	0.0208 (5)	0.0178 (5)	0.0202 (6)	−0.0027 (4)	−0.0027 (4)	0.0041 (4)
C21B	0.0223 (12)	0.0154 (8)	0.0217 (9)	−0.0054 (7)	−0.0025 (7)	0.0004 (6)
C21X	0.023 (3)	0.017 (2)	0.026 (2)	−0.0044 (18)	−0.0045 (18)	0.0040 (16)
C22B	0.0206 (6)	0.0190 (6)	0.0299 (7)	−0.0036 (4)	−0.0075 (5)	−0.0023 (5)
C23B	0.0121 (5)	0.0180 (5)	0.0232 (6)	−0.0003 (4)	−0.0040 (4)	−0.0012 (4)
C24B	0.0262 (6)	0.0182 (6)	0.0246 (6)	−0.0068 (4)	−0.0013 (5)	−0.0012 (4)

### Geometric parameters (Å, °)

**Table:**

O1A—C14A	1.3506 (14)	O4B—C11B	1.3611 (13)
O1A—C15A	1.4524 (14)	O4B—C24B	1.4331 (15)
O2A—C14A	1.2099 (14)	N1B—C7B	1.3229 (14)
O3A—C23A	1.2238 (15)	N1B—C1B	1.3883 (13)
O4A—C11A	1.3590 (13)	N2B—C6B	1.3790 (13)
O4A—C24A	1.4318 (15)	N2B—C7B	1.3847 (13)
N1A—C7A	1.3266 (14)	N2B—C17B	1.4656 (13)
N1A—C1A	1.3890 (14)	N3B—C23B	1.3519 (15)
N2A—C6A	1.3783 (13)	N3B—C19B	1.4568 (14)
N2A—C7A	1.3832 (13)	N3B—C20B	1.4608 (15)
N2A—C17A	1.4651 (13)	C1B—C2B	1.3939 (15)
N3A—C23A	1.3550 (15)	C1B—C6B	1.4109 (14)
N3A—C19A	1.4565 (14)	C2B—C3B	1.3920 (15)
N3A—C20A	1.4579 (15)	C2B—H2BA	0.9500
C1A—C2A	1.3957 (15)	C3B—C4B	1.4147 (15)
C1A—C6A	1.4101 (14)	C3B—C14B	1.4851 (15)
C2A—C3A	1.3968 (15)	C4B—C5B	1.3832 (15)
C2A—H2AA	0.9500	C4B—H4BA	0.9500
C3A—C4A	1.4120 (15)	C5B—C6B	1.3941 (14)
C3A—C14A	1.4861 (15)	C5B—H5BA	0.9500
C4A—C5A	1.3857 (15)	C7B—C8B	1.4698 (15)
C4A—H4AA	0.9500	C8B—C9B	1.3908 (15)
C5A—C6A	1.3941 (14)	C8B—C13B	1.4044 (15)
C5A—H5AA	0.9500	C9B—C10B	1.3958 (15)
C7A—C8A	1.4718 (15)	C9B—H9BA	0.9500
C8A—C9A	1.3954 (15)	C10B—C11B	1.3955 (15)
C8A—C13A	1.4083 (15)	C10B—H10B	0.9500
C9A—C10A	1.3955 (15)	C11B—C12B	1.4012 (16)
C9A—H9AA	0.9500	C12B—C13B	1.3817 (16)
C10A—C11A	1.3920 (15)	C12B—H12B	0.9500
C10A—H10A	0.9500	C13B—H13B	0.9500
C11A—C12A	1.4001 (16)	C15B—C16B	1.4991 (19)
C12A—C13A	1.3813 (16)	C15B—H15C	0.9900
C12A—H12A	0.9500	C15B—H15D	0.9900
C13A—H13A	0.9500	C16B—H16D	0.9800
C15A—C16A	1.500 (2)	C16B—H16E	0.9800
C15A—H15A	0.9900	C16B—H16F	0.9800
C15A—H15B	0.9900	C17B—C18B	1.5269 (15)
C16A—H16A	0.9800	C17B—H17C	0.9900
C16A—H16B	0.9800	C17B—H17D	0.9900
C16A—H16C	0.9800	C18B—C19B	1.5283 (14)
C17A—C18A	1.5279 (15)	C18B—H18C	0.9900
C17A—H17A	0.9900	C18B—H18D	0.9900
C17A—H17B	0.9900	C19B—H19C	0.9900
C18A—C19A	1.5248 (15)	C19B—H19D	0.9900
C18A—H18A	0.9900	C20B—C21X	1.462 (4)
C18A—H18B	0.9900	C20B—C21B	1.565 (2)
C19A—H19A	0.9900	C20B—H20C	0.9900
C19A—H19B	0.9900	C20B—H20D	0.9900
C20A—C21A	1.5282 (18)	C20B—H20E	0.9600
C20A—H20A	0.9900	C20B—H20F	0.9600
C20A—H20B	0.9900	C21B—C22B	1.477 (2)
C21A—C22A	1.5181 (19)	C21B—H21C	0.9900
C21A—H21A	0.9900	C21B—H21D	0.9900
C21A—H21B	0.9900	C21X—C22B	1.652 (5)
C22A—C23A	1.5193 (16)	C21X—H21E	0.9900
C22A—H22A	0.9900	C21X—H21F	0.9900
C22A—H22B	0.9900	C22B—C23B	1.5209 (16)
C24A—H24A	0.9800	C22B—H22C	0.9900
C24A—H24B	0.9800	C22B—H22D	0.9900
C24A—H24C	0.9800	C22B—H22E	0.9601
O1B—C14B	1.3490 (14)	C22B—H22F	0.9599
O1B—C15B	1.4560 (14)	C24B—H24D	0.9800
O2B—C14B	1.2099 (14)	C24B—H24E	0.9800
O3B—C23B	1.2255 (15)	C24B—H24F	0.9800
			
C14A—O1A—C15A	116.38 (10)	C2B—C3B—C4B	121.21 (10)
C11A—O4A—C24A	118.17 (9)	C2B—C3B—C14B	117.62 (10)
C7A—N1A—C1A	104.81 (9)	C4B—C3B—C14B	121.05 (10)
C6A—N2A—C7A	106.44 (9)	C5B—C4B—C3B	121.62 (10)
C6A—N2A—C17A	123.00 (9)	C5B—C4B—H4BA	119.2
C7A—N2A—C17A	130.56 (9)	C3B—C4B—H4BA	119.2
C23A—N3A—C19A	123.60 (10)	C4B—C5B—C6B	116.45 (10)
C23A—N3A—C20A	112.69 (10)	C4B—C5B—H5BA	121.8
C19A—N3A—C20A	121.86 (10)	C6B—C5B—H5BA	121.8
N1A—C1A—C2A	130.19 (10)	N2B—C6B—C5B	131.51 (10)
N1A—C1A—C6A	110.04 (9)	N2B—C6B—C1B	105.57 (9)
C2A—C1A—C6A	119.77 (10)	C5B—C6B—C1B	122.92 (10)
C1A—C2A—C3A	118.01 (10)	N1B—C7B—N2B	113.22 (9)
C1A—C2A—H2AA	121.0	N1B—C7B—C8B	123.28 (9)
C3A—C2A—H2AA	121.0	N2B—C7B—C8B	123.51 (9)
C2A—C3A—C4A	120.96 (10)	C9B—C8B—C13B	118.28 (10)
C2A—C3A—C14A	117.88 (10)	C9B—C8B—C7B	122.23 (9)
C4A—C3A—C14A	121.14 (10)	C13B—C8B—C7B	119.42 (10)
C5A—C4A—C3A	121.85 (10)	C8B—C9B—C10B	121.81 (10)
C5A—C4A—H4AA	119.1	C8B—C9B—H9BA	119.1
C3A—C4A—H4AA	119.1	C10B—C9B—H9BA	119.1
C4A—C5A—C6A	116.44 (10)	C11B—C10B—C9B	119.00 (10)
C4A—C5A—H5AA	121.8	C11B—C10B—H10B	120.5
C6A—C5A—H5AA	121.8	C9B—C10B—H10B	120.5
N2A—C6A—C5A	131.32 (10)	O4B—C11B—C10B	124.63 (10)
N2A—C6A—C1A	105.72 (9)	O4B—C11B—C12B	115.51 (10)
C5A—C6A—C1A	122.95 (10)	C10B—C11B—C12B	119.86 (10)
N1A—C7A—N2A	112.99 (10)	C13B—C12B—C11B	120.26 (10)
N1A—C7A—C8A	122.22 (10)	C13B—C12B—H12B	119.9
N2A—C7A—C8A	124.79 (10)	C11B—C12B—H12B	119.9
C9A—C8A—C13A	117.70 (10)	C12B—C13B—C8B	120.74 (11)
C9A—C8A—C7A	124.36 (10)	C12B—C13B—H13B	119.6
C13A—C8A—C7A	117.85 (10)	C8B—C13B—H13B	119.6
C10A—C9A—C8A	121.69 (10)	O2B—C14B—O1B	123.42 (10)
C10A—C9A—H9AA	119.2	O2B—C14B—C3B	124.53 (11)
C8A—C9A—H9AA	119.2	O1B—C14B—C3B	112.02 (9)
C11A—C10A—C9A	119.53 (10)	O1B—C15B—C16B	110.85 (11)
C11A—C10A—H10A	120.2	O1B—C15B—H15C	109.5
C9A—C10A—H10A	120.2	C16B—C15B—H15C	109.5
O4A—C11A—C10A	124.98 (10)	O1B—C15B—H15D	109.5
O4A—C11A—C12A	115.35 (10)	C16B—C15B—H15D	109.5
C10A—C11A—C12A	119.66 (10)	H15C—C15B—H15D	108.1
C13A—C12A—C11A	120.20 (10)	C15B—C16B—H16D	109.5
C13A—C12A—H12A	119.9	C15B—C16B—H16E	109.5
C11A—C12A—H12A	119.9	H16D—C16B—H16E	109.5
C12A—C13A—C8A	121.19 (11)	C15B—C16B—H16F	109.5
C12A—C13A—H13A	119.4	H16D—C16B—H16F	109.5
C8A—C13A—H13A	119.4	H16E—C16B—H16F	109.5
O2A—C14A—O1A	123.37 (11)	N2B—C17B—C18B	111.97 (9)
O2A—C14A—C3A	124.80 (11)	N2B—C17B—H17C	109.2
O1A—C14A—C3A	111.83 (10)	C18B—C17B—H17C	109.2
O1A—C15A—C16A	110.50 (11)	N2B—C17B—H17D	109.2
O1A—C15A—H15A	109.5	C18B—C17B—H17D	109.2
C16A—C15A—H15A	109.5	H17C—C17B—H17D	107.9
O1A—C15A—H15B	109.5	C17B—C18B—C19B	112.42 (9)
C16A—C15A—H15B	109.5	C17B—C18B—H18C	109.1
H15A—C15A—H15B	108.1	C19B—C18B—H18C	109.1
C15A—C16A—H16A	109.5	C17B—C18B—H18D	109.1
C15A—C16A—H16B	109.5	C19B—C18B—H18D	109.1
H16A—C16A—H16B	109.5	H18C—C18B—H18D	107.9
C15A—C16A—H16C	109.5	N3B—C19B—C18B	113.49 (9)
H16A—C16A—H16C	109.5	N3B—C19B—H19C	108.9
H16B—C16A—H16C	109.5	C18B—C19B—H19C	108.9
N2A—C17A—C18A	111.28 (9)	N3B—C19B—H19D	108.9
N2A—C17A—H17A	109.4	C18B—C19B—H19D	108.9
C18A—C17A—H17A	109.4	H19C—C19B—H19D	107.7
N2A—C17A—H17B	109.4	N3B—C20B—C21X	108.55 (19)
C18A—C17A—H17B	109.4	N3B—C20B—C21B	101.86 (10)
H17A—C17A—H17B	108.0	N3B—C20B—H20C	111.4
C19A—C18A—C17A	112.21 (9)	C21X—C20B—H20C	80.0
C19A—C18A—H18A	109.2	C21B—C20B—H20C	111.4
C17A—C18A—H18A	109.2	N3B—C20B—H20D	111.4
C19A—C18A—H18B	109.2	C21X—C20B—H20D	131.3
C17A—C18A—H18B	109.2	C21B—C20B—H20D	111.4
H18A—C18A—H18B	107.9	H20C—C20B—H20D	109.3
N3A—C19A—C18A	112.73 (9)	N3B—C20B—H20E	110.0
N3A—C19A—H19A	109.0	C21X—C20B—H20E	110.0
C18A—C19A—H19A	109.0	C21B—C20B—H20E	138.5
N3A—C19A—H19B	109.0	H20D—C20B—H20E	81.2
C18A—C19A—H19B	109.0	N3B—C20B—H20F	110.0
H19A—C19A—H19B	107.8	C21X—C20B—H20F	110.0
N3A—C20A—C21A	102.67 (10)	C21B—C20B—H20F	83.9
N3A—C20A—H20A	111.2	H20C—C20B—H20F	131.2
C21A—C20A—H20A	111.2	H20E—C20B—H20F	108.4
N3A—C20A—H20B	111.2	C22B—C21B—C20B	104.43 (12)
C21A—C20A—H20B	111.2	C22B—C21B—H21C	110.9
H20A—C20A—H20B	109.1	C20B—C21B—H21C	110.9
C22A—C21A—C20A	103.30 (10)	C22B—C21B—H21D	110.9
C22A—C21A—H21A	111.1	C20B—C21B—H21D	110.9
C20A—C21A—H21A	111.1	H21C—C21B—H21D	108.9
C22A—C21A—H21B	111.1	C20B—C21X—C22B	101.0 (3)
C20A—C21A—H21B	111.1	C20B—C21X—H21E	111.6
H21A—C21A—H21B	109.1	C22B—C21X—H21E	111.6
C21A—C22A—C23A	103.75 (10)	C20B—C21X—H21F	111.6
C21A—C22A—H22A	111.0	C22B—C21X—H21F	111.6
C23A—C22A—H22A	111.0	H21E—C21X—H21F	109.4
C21A—C22A—H22B	111.0	C21B—C22B—C23B	104.81 (11)
C23A—C22A—H22B	111.0	C23B—C22B—C21X	103.05 (17)
H22A—C22A—H22B	109.0	C21B—C22B—H22C	110.8
O3A—C23A—N3A	125.52 (11)	C23B—C22B—H22C	110.8
O3A—C23A—C22A	126.59 (11)	C21X—C22B—H22C	83.6
N3A—C23A—C22A	107.88 (10)	C21B—C22B—H22D	110.8
O4A—C24A—H24A	109.5	C23B—C22B—H22D	110.8
O4A—C24A—H24B	109.5	C21X—C22B—H22D	135.8
H24A—C24A—H24B	109.5	H22C—C22B—H22D	108.9
O4A—C24A—H24C	109.5	C21B—C22B—H22E	82.5
H24A—C24A—H24C	109.5	C23B—C22B—H22E	111.1
H24B—C24A—H24C	109.5	C21X—C22B—H22E	111.1
C14B—O1B—C15B	116.14 (9)	H22C—C22B—H22E	130.4
C11B—O4B—C24B	118.21 (9)	C21B—C22B—H22F	133.9
C7B—N1B—C1B	104.69 (9)	C23B—C22B—H22F	111.2
C6B—N2B—C7B	106.31 (9)	C21X—C22B—H22F	111.2
C6B—N2B—C17B	124.17 (9)	H22D—C22B—H22F	82.4
C7B—N2B—C17B	129.50 (9)	H22E—C22B—H22F	109.1
C23B—N3B—C19B	123.55 (10)	O3B—C23B—N3B	125.50 (11)
C23B—N3B—C20B	113.26 (9)	O3B—C23B—C22B	126.40 (11)
C19B—N3B—C20B	123.16 (10)	N3B—C23B—C22B	108.10 (10)
N1B—C1B—C2B	129.97 (10)	O4B—C24B—H24D	109.5
N1B—C1B—C6B	110.21 (9)	O4B—C24B—H24E	109.5
C2B—C1B—C6B	119.77 (10)	H24D—C24B—H24E	109.5
C3B—C2B—C1B	117.91 (10)	O4B—C24B—H24F	109.5
C3B—C2B—H2BA	121.0	H24D—C24B—H24F	109.5
C1B—C2B—H2BA	121.0	H24E—C24B—H24F	109.5
			
C7A—N1A—C1A—C2A	178.81 (12)	C1B—C2B—C3B—C14B	−174.69 (10)
C7A—N1A—C1A—C6A	−0.57 (12)	C2B—C3B—C4B—C5B	−2.86 (17)
N1A—C1A—C2A—C3A	179.72 (11)	C14B—C3B—C4B—C5B	173.15 (10)
C6A—C1A—C2A—C3A	−0.95 (16)	C3B—C4B—C5B—C6B	1.04 (16)
C1A—C2A—C3A—C4A	−0.36 (17)	C7B—N2B—C6B—C5B	−179.16 (11)
C1A—C2A—C3A—C14A	178.38 (10)	C17B—N2B—C6B—C5B	−0.53 (18)
C2A—C3A—C4A—C5A	1.03 (17)	C7B—N2B—C6B—C1B	0.57 (11)
C14A—C3A—C4A—C5A	−177.67 (11)	C17B—N2B—C6B—C1B	179.20 (9)
C3A—C4A—C5A—C6A	−0.34 (16)	C4B—C5B—C6B—N2B	−178.22 (11)
C7A—N2A—C6A—C5A	179.04 (11)	C4B—C5B—C6B—C1B	2.09 (16)
C17A—N2A—C6A—C5A	−0.97 (18)	N1B—C1B—C6B—N2B	−0.91 (12)
C7A—N2A—C6A—C1A	−0.24 (11)	C2B—C1B—C6B—N2B	176.76 (10)
C17A—N2A—C6A—C1A	179.75 (9)	N1B—C1B—C6B—C5B	178.85 (10)
C4A—C5A—C6A—N2A	179.80 (11)	C2B—C1B—C6B—C5B	−3.48 (16)
C4A—C5A—C6A—C1A	−1.02 (16)	C1B—N1B—C7B—N2B	−0.52 (12)
N1A—C1A—C6A—N2A	0.51 (12)	C1B—N1B—C7B—C8B	179.13 (10)
C2A—C1A—C6A—N2A	−178.94 (10)	C6B—N2B—C7B—N1B	−0.04 (12)
N1A—C1A—C6A—C5A	−178.85 (10)	C17B—N2B—C7B—N1B	−178.57 (10)
C2A—C1A—C6A—C5A	1.70 (17)	C6B—N2B—C7B—C8B	−179.69 (10)
C1A—N1A—C7A—N2A	0.43 (12)	C17B—N2B—C7B—C8B	1.78 (17)
C1A—N1A—C7A—C8A	179.73 (10)	N1B—C7B—C8B—C9B	−128.34 (12)
C6A—N2A—C7A—N1A	−0.12 (12)	N2B—C7B—C8B—C9B	51.28 (16)
C17A—N2A—C7A—N1A	179.90 (10)	N1B—C7B—C8B—C13B	48.53 (16)
C6A—N2A—C7A—C8A	−179.40 (10)	N2B—C7B—C8B—C13B	−131.85 (12)
C17A—N2A—C7A—C8A	0.61 (18)	C13B—C8B—C9B—C10B	1.75 (17)
N1A—C7A—C8A—C9A	142.34 (12)	C7B—C8B—C9B—C10B	178.66 (10)
N2A—C7A—C8A—C9A	−38.44 (17)	C8B—C9B—C10B—C11B	0.26 (17)
N1A—C7A—C8A—C13A	−34.16 (16)	C24B—O4B—C11B—C10B	−0.90 (16)
N2A—C7A—C8A—C13A	145.06 (11)	C24B—O4B—C11B—C12B	179.28 (11)
C13A—C8A—C9A—C10A	−0.77 (17)	C9B—C10B—C11B—O4B	178.28 (10)
C7A—C8A—C9A—C10A	−177.28 (10)	C9B—C10B—C11B—C12B	−1.91 (17)
C8A—C9A—C10A—C11A	−0.42 (17)	O4B—C11B—C12B—C13B	−178.67 (11)
C24A—O4A—C11A—C10A	0.81 (17)	C10B—C11B—C12B—C13B	1.50 (18)
C24A—O4A—C11A—C12A	179.96 (11)	C11B—C12B—C13B—C8B	0.57 (19)
C9A—C10A—C11A—O4A	−179.57 (11)	C9B—C8B—C13B—C12B	−2.17 (17)
C9A—C10A—C11A—C12A	1.32 (17)	C7B—C8B—C13B—C12B	−179.17 (11)
O4A—C11A—C12A—C13A	179.80 (11)	C15B—O1B—C14B—O2B	1.87 (17)
C10A—C11A—C12A—C13A	−1.00 (18)	C15B—O1B—C14B—C3B	−176.53 (10)
C11A—C12A—C13A—C8A	−0.22 (19)	C2B—C3B—C14B—O2B	13.21 (17)
C9A—C8A—C13A—C12A	1.09 (17)	C4B—C3B—C14B—O2B	−162.95 (12)
C7A—C8A—C13A—C12A	177.83 (11)	C2B—C3B—C14B—O1B	−168.41 (10)
C15A—O1A—C14A—O2A	−1.09 (18)	C4B—C3B—C14B—O1B	15.43 (15)
C15A—O1A—C14A—C3A	178.91 (10)	C14B—O1B—C15B—C16B	−81.55 (14)
C2A—C3A—C14A—O2A	−1.66 (18)	C6B—N2B—C17B—C18B	75.69 (13)
C4A—C3A—C14A—O2A	177.08 (12)	C7B—N2B—C17B—C18B	−106.01 (12)
C2A—C3A—C14A—O1A	178.34 (10)	N2B—C17B—C18B—C19B	−178.22 (9)
C4A—C3A—C14A—O1A	−2.92 (16)	C23B—N3B—C19B—C18B	122.57 (12)
C14A—O1A—C15A—C16A	85.97 (14)	C20B—N3B—C19B—C18B	−59.60 (14)
C6A—N2A—C17A—C18A	−77.35 (12)	C17B—C18B—C19B—N3B	−62.93 (12)
C7A—N2A—C17A—C18A	102.63 (12)	C23B—N3B—C20B—C21X	−14.2 (3)
N2A—C17A—C18A—C19A	179.58 (9)	C19B—N3B—C20B—C21X	167.8 (3)
C23A—N3A—C19A—C18A	−119.70 (12)	C23B—N3B—C20B—C21B	18.41 (14)
C20A—N3A—C19A—C18A	76.91 (13)	C19B—N3B—C20B—C21B	−159.62 (12)
C17A—C18A—C19A—N3A	64.97 (12)	N3B—C20B—C21B—C22B	−26.58 (16)
C23A—N3A—C20A—C21A	22.69 (14)	C21X—C20B—C21B—C22B	79.5 (4)
C19A—N3A—C20A—C21A	−172.26 (10)	N3B—C20B—C21X—C22B	22.6 (3)
N3A—C20A—C21A—C22A	−30.40 (13)	C21B—C20B—C21X—C22B	−60.1 (3)
C20A—C21A—C22A—C23A	27.87 (13)	C20B—C21B—C22B—C23B	25.41 (16)
C19A—N3A—C23A—O3A	8.94 (18)	C20B—C21B—C22B—C21X	−65.3 (3)
C20A—N3A—C23A—O3A	173.68 (11)	C20B—C21X—C22B—C21B	73.6 (4)
C19A—N3A—C23A—C22A	−169.69 (10)	C20B—C21X—C22B—C23B	−23.5 (3)
C20A—N3A—C23A—C22A	−4.94 (13)	C19B—N3B—C23B—O3B	−4.92 (18)
C21A—C22A—C23A—O3A	166.27 (12)	C20B—N3B—C23B—O3B	177.06 (11)
C21A—C22A—C23A—N3A	−15.13 (13)	C19B—N3B—C23B—C22B	175.12 (10)
C7B—N1B—C1B—C2B	−176.49 (11)	C20B—N3B—C23B—C22B	−2.90 (13)
C7B—N1B—C1B—C6B	0.88 (12)	C21B—C22B—C23B—O3B	164.98 (14)
N1B—C1B—C2B—C3B	178.73 (11)	C21X—C22B—C23B—O3B	−163.5 (2)
C6B—C1B—C2B—C3B	1.58 (16)	C21B—C22B—C23B—N3B	−15.06 (15)
C1B—C2B—C3B—C4B	1.46 (16)	C21X—C22B—C23B—N3B	16.5 (2)

### Hydrogen-bond geometry (Å, °)

**Table:**

Cg1, Cg2, Cg4, Cg5 and Cg6 are the centroids of the N1*B*–N2*B*/C1*B*/C6*B*–C7*B*, C1*A*–C6*A*, C1*B*–C6*B*, C8*B*–C13*B* and C8*A*–C13*A* rings, respectively.

**Table:**

*D*—H···*A*	*D*—H	H···*A*	*D*···*A*	*D*—H···*A*
C5A—H5AA···O3B^i^	0.95	2.30	3.2382 (15)	171
C5B—H5BA···O3A^ii^	0.95	2.38	3.3244 (15)	172
C19A—H19A···O2A^i^	0.99	2.58	3.3034 (14)	130
C19A—H19B···O3B^i^	0.99	2.53	3.2681 (15)	131
C19B—H19C···O3A^ii^	0.99	2.46	3.2007 (15)	131
C9B—H9BA···Cg1^iii^	0.95	2.85	3.5237 (12)	129
C10B—H10B···Cg4^iii^	0.95	2.80	3.4556 (12)	127
C16A—H16B···Cg5	0.98	2.83	3.8093 (15)	175
C18A—H18A···Cg6^iv^	0.99	2.70	3.5251 (12)	141
C24A—H24C···Cg2^iv^	0.98	2.76	3.7186 (14)	165

Symmetry codes: (i) *x*−1, *y*, *z*; (ii) *x*+1, *y*, *z*; (iii) −*x*+1, −*y*+1, −*z*; (iv) −*x*, −*y*, −*z*+1.
